# Enhancing the Quality and Nutritional Properties of Gluten‐Free Pancakes Using Sprouted Quinoa Flour Treated With Magnetic Fields, Ultrasound, and Infrared Drying

**DOI:** 10.1002/fsn3.70502

**Published:** 2025-07-18

**Authors:** Sepideh Vejdanivahid, Fakhreddin Salehi

**Affiliations:** ^1^ Department of Food Science and Technology, Faculty of Food Industry Bu‐Ali Sina University Hamedan Iran

**Keywords:** baking loss, gluten‐free, sensory attributes, texture, total phenolic content

## Abstract

Quinoa seed flour can be utilized to make gluten‐free pancakes. Enhancing the quality and nutritional value of sprouted quinoa flour can be achieved through the application of magnetic fields, ultrasound, and infrared drying techniques. So, in this study, wheat, quinoa seeds (unsprouted), and sprouted quinoa seeds flours were used to prepare pancakes. Then, the pH, acidity, color indexes, total phenolic content, antioxidant capacity, baking loss, volume, density, hardness, and sensory properties of pancakes were evaluated and compared. The pancake batter made from sprouted quinoa flour was thicker than (higher viscosity) the other batters and had a darker and more yellow color. Sprouting reduces the pH and increases the acidity of sprouted quinoa flour, so the pH and acidity of pancakes made from it were lower and higher than those of other pancakes, respectively. By replacing sprouted quinoa flour in the formulation, the crust color of the pancake became darker and redder, but the yellowness index decreased. Pancakes prepared from sprouted quinoa flour exhibited considerably increased levels of both total phenolic content and antioxidant capacity, indicating an enhancement in these nutritional parameters. These pancakes had the lowest baking loss, density, and hardness compared to other samples. In most sensory attributes, especially overall acceptance, pancakes made with sprouted quinoa flour received the highest scores, whereas pancakes made with unsprouted quinoa flour received the lowest scores. In general, because it is very nutritious and has a good taste and texture, sprouted quinoa flour is a good choice for making gluten‐free food products.

## Graphical abstract

The incorporation of sprouted quinoa flour in pancake formulation enhanced nutritional and quality attributes, resulting in increased phenolic content and antioxidant capacity. This pancake also exhibited the lowest values for baking loss, density, and hardness. Sensory analysis indicated the highest overall acceptance, highlighting its potential as a valuable ingredient in gluten‐free products.

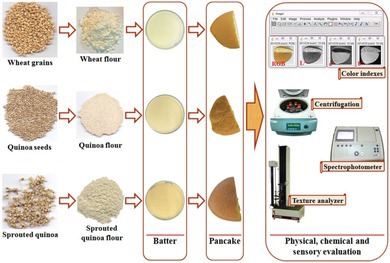

## 1 Introduction

Cereals and pseudocereals constitute fundamental components of the human diet, serving as primary sources of essential nutrients and playing a critical role in global food security (Estivi et al. 2022). Quinoa ( *Chenopodium quinoa* Willd.) is a highly nutritious pseudocereal that has attracted worldwide attention due to its exceptional nutritional profile and adaptability to diverse growing conditions. Native to the Andean region of South America, quinoa is rich in high quality protein, contains all nine essential amino acids, and is also a good source of dietary fiber, vitamins, and minerals (Castro‐Alba et al. 2019; Olivera‐Montenegro et al. 2021; Arguello‐Hernández et al. 2024; Khan et al. 2025). In contrast to many grains, quinoa is gluten‐free, making it a suitable option for people with gluten intolerance or celiac disease. Additionally, quinoa contains a variety of bioactive compounds, including flavonoids and phenolic acids, which contribute to its antioxidant capacity. Its resilience to harsh environmental conditions and its health‐promoting ingredients make quinoa an important crop for both food security and functional food development (Vejdanivahid and Salehi 2024; Davtalab et al. 2025; Moradnia et al. 2025).

Sprouting is a biological process that activates enzymatic activity in grains, leading to the breakdown of antinutritional factors and the enhancement of their nutritional and functional properties (Salehi 2023a; Yılmaz Tuncel et al. 2025). During sprouting, complex compounds such as starches and proteins are partially hydrolyzed into simpler, more digestible forms, improving the bioavailability of essential nutrients including vitamins (especially B complex), minerals, and amino acids. Additionally, sprouted grains exhibit increased levels of bioactive compounds, such as phenolic compounds and antioxidants, which contribute to potential health benefits (Amin Ekhlas et al. 2024; Goharpour et al. 2025; Yılmaz Tuncel et al. 2025). Incorporating sprouted grains into food formulations has been shown to improve the sensory qualities, nutritional value, and health‐promoting properties of various products, such as breads, cakes, biscuits, cupcakes, and baked goods. This makes sprouted grains an attractive ingredient in the development of functional and health‐oriented foods (Suárez‐Estrella et al. 2020; Paucar‐Menacho et al. 2022; Amin Ekhlas et al. 2023; Goharpour et al. 2024; Marie et al. 2024). According to Suárez‐Estrella et al. (2020), sprouting represents an effective strategy for the development of quinoa‐enriched bread, aimed at promoting the production and consumption of fiber‐rich products alongside proteins of high biological value.

Magnetized water has shown promising benefits in enhancing the growth and nutritional value of cereal crops. By altering the physical and chemical properties of water, magnetization improves water absorption and nutrient uptake in plants, leading to accelerated germination, increased biomass, and higher yields. Additionally, cereals irrigated with magnetized water often exhibit improved protein content, enhanced enzymatic activity, and better overall nutritional profiles. This eco‐friendly and cost‐effective method supports sustainable agriculture by reducing the need for chemical fertilizers and promoting healthier crop development (Aliverdi et al. 2021; Al‐Akhras et al. 2024; Chen et al. 2025).

Ultrasound pretreatment has emerged as an effective technique to enhance the nutritional quality and functional properties of cereal grains (Estivi et al. 2022; Amin Ekhlas et al. 2024). By applying high frequency sound waves, this method disrupts cellular structures, increases enzymatic activity, and facilitates the release of bioactive compounds. As a result, cereals subjected to ultrasound treatment often exhibit improved protein digestibility, enhanced antioxidant activity, and greater bioavailability of essential nutrients such as vitamins and minerals. This nonthermal and environmentally friendly approach contributes to producing healthier cereal‐based foods while preserving their natural quality (Estivi et al. 2022; Salehi 2023b; Khan et al. 2025). Estivi et al. (2022) investigated the effects of low frequency ultrasound on the physical, chemical, and technological characteristics of cereals and pseudocereals. Their findings highlighted the technology's potential to enhance processing efficiency and functional characteristics by promoting the extraction of bioactive compounds, altering starch structures, and partially denaturing proteins to improve their functionality and bioavailability.

Infrared drying technology offers several advantages for drying agricultural products, making it an efficient and sustainable alternative to conventional methods. This technique provides rapid and uniform heat transfer, significantly reducing drying time and energy consumption. Infrared drying also minimizes nutrient loss, preserves the color, texture, and flavor of products, and reduces microbial contamination due to its effective surface heating. As a result, it enhances the overall quality and shelf life of dried agricultural goods while supporting eco‐friendly processing practices (Salehi 2020; Goharpour et al. 2024; Stan et al. 2025).

Celiac disease is a lifelong intestinal disease caused by environmental and genetic factors, and as a result, the consumption of gluten protein in these individuals damages the mucous membrane of the small intestine and reduces the absorption of essential nutrients such as proteins, carbohydrates, fat‐soluble vitamins, iron, and calcium. The only way to treat this disease is to use a gluten‐free diet for life. Therefore, the production of gluten‐free products is one of the priorities of the food industry to help these people (Jnawali et al. 2016; Salehi 2019b). A pancake is a flat cake, often thin and round, that is often made by mixing wheat flour, eggs, milk, and butter or oil with baking powder and is used as a breakfast or snack. This snack is very popular among people, especially children (Kiprushkina et al. 2020; Rakmai et al. 2021).

It is essential to investigate the potential of quinoa flour as a substitute for wheat flour in the formulation of gluten‐free pancakes, offering a nutritionally rich snack alternative. Moreover, the nutritional quality of quinoa sprouts can be further improved through the use of magnetized water, ultrasound treatment, and infrared drying, which are innovative and sustainable processing techniques known to improve the bioavailability of nutrients and functional properties of food products. So, in this study, wheat, quinoa seeds (unsprouted), and sprouted quinoa seeds flours were used to prepare pancakes, and the physical, chemical, textural, and sensorial characteristics of products were evaluated. In addition, the color indexes and viscosity of pancake batter were measured.

## 2 Materials and Methods

### 2.1 Preparing Sprouted Quinoa

We purchased white quinoa seeds harvested from Iran (packaged by OAB Company, Iran). First, the quinoa seeds were cleaned and soaked in magnetized water in a magnetic field for 1 h at 25°C. Then, the seeds were poured into a flat container and covered with a thin towel. During the sprouting stage, the seeds were placed entirely inside the magnetic field along with the container and towel. The seeds were moistened with a water sprayer bottle every 6 h. In total, the seeds were kept at a temperature of about 25°C for 72 h until they sprouted (Vejdanivahid and Salehi 2024).

A magnetic‐alkaline ionized water production device (bipolar model with timer, Meghnatis Sazan Hayat Co., Iran) was used to magnetize the water and prepare the magnetized water. The strength of the magnetic field created by the device was checked with a Gauss meter (Model TES‐3196, Taiwan). The device created a magnetic field strength of 2.8 G and the magnetic field strength of the magnetized water inside the device was 1.4 G.

### 2.2 Ultrasonic Pretreatment

Ultrasonic pretreatment was used on the sprouted quinoa seeds with an ultrasonic water bath (Backer, vCLEAN1‐L6, Iran). This device holds 6 L of water and works at a frequency of 40 kHz with a power of 150 watts. The sprouted quinoa seeds were put in the ultrasonic water bath at 25°C for 5 min.

### 2.3 Drying of Sprouted Quinoa

To dry the ultrasonic‐pretreated sprouted seeds, the quinoa sprouts were placed in an infrared dryer (250 W, near‐infrared, Iran). The weight of the quinoa sprouts was measured every 1 min using a digital balance (GM‐300p, Lutron, Taiwan) with a precision of 0.01 g until a constant weight was reached.

### 2.4 Powdering the Dried Sprouts

Dried quinoa sprouts were ground using an industrial grinder (Best, China). The prepared flour was packed into polyethylene bags to prevent moisture absorption during storage. The bags were then stored in a refrigerator at 6°C until the experiment.

### 2.5 Pancake Preparation

**FIGURE 1:**
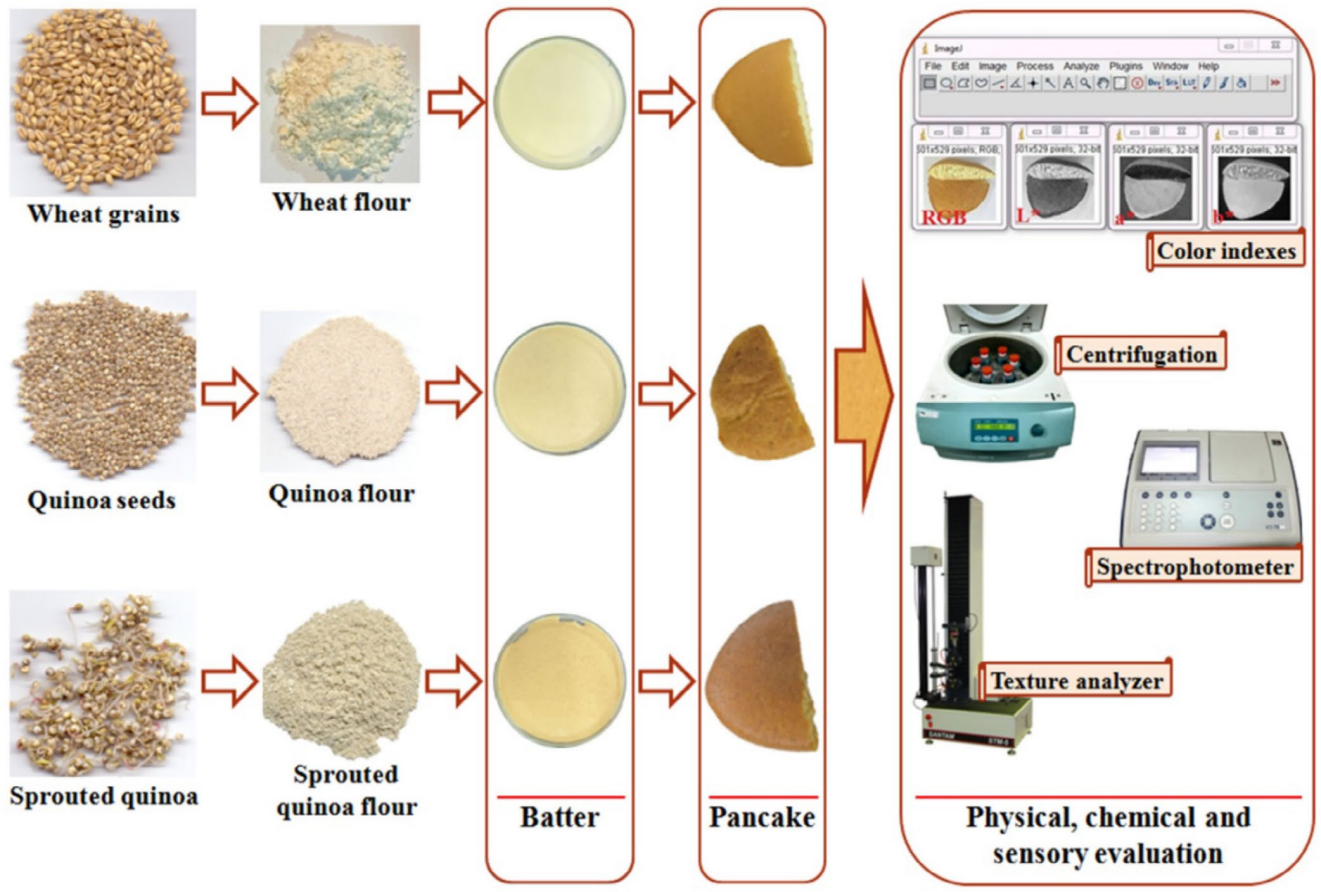
Steps of pancake preparation using wheat, quinoa seed, and sprouted quinoa seed flours.

In this study, wheat flour (Zar macaron, Iran), quinoa seeds flour (unsprouted), and sprouted quinoa seeds flour (flour prepared from previous steps) were used to prepare pancakes (Figure 1).

After reviewing published articles and trial and error, a suitable formulation for making pancakes was determined, which included 70 g of flour (26.6%), 1 g of vanilla (0.4%), 4 g of baking powder (1.5%), 6 g of oil (2.3%), 25 g of sugar (9.5%), 100 g of milk (38.0%), and 57 g of egg (21.7%). For this study, baking powder (Bartar, Iran), vanilla (Golha, Golha Food and Agriculture Complex, Iran), low‐fat milk (Pegah, Iran), frying oil (Famila, Iran), sugar (Iran), and fresh eggs were also purchased from a store in Hamedan, Iran.

First, the dry ingredients were mixed and sieved. The eggs were beaten with an electric mixer for 3 min. The liquid ingredients (milk and oil) were added and mixed. The dry ingredients were added to the mixture and mixing continued for 5 min. The prepared batter was allowed to rest at room temperature for 10 min. For baking, the pan was heated to 170°C–180°C, and 25 g of batter was baked in the pan for 2 min until bubbles appeared on the surface of the pancake, then the other side of the pancake was baked for another 1 min. After cooling, the baked pancakes were packaged and stored in moisture and oxygen resistant polyethylene containers. The cooking temperature was measured with a digital thermometer.

### 2.6 Evaluation of Batter Color and Viscosity

Image processing methods were used to measure the pancake batter color indexes. The batter photos were captured with a 48‐megapixel camera (iPhone 15 Pro Max; Apple Co., China). The color space of the photos was converted from RGB to L* (lightness), a* (green/red), and b* (blue/yellow) using ImageJ software (V.1.42e, USA) and the corresponding plugin (Salehi 2019a).

The apparent viscosity changes of pancake batter as a function of spindle revolution speed (5, 10, and 15 RMP) and time (0–120 s) were measured using a rotational viscometer (Brookfield, DV2T, RV, USA) by spindle RV‐02 at 25°C.

### 2.7 pH and Acidity of Pancakes

Based on the Iranian National Standard No. 37 (2018), the pH of the pancakes was determined by homogenizing 10 g of sample with 100 mL of distilled water and allowing it to stand for 20 min. Following the calibration of the pH meter (Metrohm, 827 pH Lab, Switzerland) using buffer solutions at pH 4 and 7, the pH of the resulting mixture was measured.

Based on Iranian National Standard No. 103 (2018), the acidity of the pancake samples was determined by extracting 10 g of the sample with 50 mL of 67% ethanol. After stirring and filtration, 25 mL of the filtrate was titrated with 0.1 N NaOH using 3% phenolphthalein as an indicator. The volume of NaOH required to reach the pink endpoint was recorded, and acidity was calculated according to the method outlined by Amin Ekhlas et al. (2024).

### 2.8 Color Parameters of Pancakes

Image processing techniques were employed to assess the color parameters of the pancake core and crust. Images were captured using a 48‐megapixel camera (iPhone 15 Pro Max, Apple Co., China), and the RGB color space was converted to L*, a*, and b* values using ImageJ software (v1.42e, USA) with a dedicated plugin (Salehi 2019a).

### 2.9 Total Phenolic Content of Pancakes

The total phenolic content of pancakes was measured using the method explained by Amin Ekhlas et al. (2024). The total phenolic content of pancakes was expressed as microgram gallic acid equivalent per g (μg GAE/g).

### 2.10 Antioxidant Capacity of Pancakes

The antioxidant capacity of the pancakes was evaluated using the DPPH assay, following the method of Salehi et al. (2023). A 0.1 mM DPPH solution (2,2‐diphenyl‐1‐picrylhydrazyl; Sigma‐Aldrich, USA) was prepared, and pancake extracts were obtained by mixing 2 g of sample with 20 mL of 80% methanol for 30 min using a magnetic stirrer (Shimaz, Iran). The mixture was then centrifuged at 4000 rpm for 5 min (Universal 320R; Hettich, Germany), and the supernatant was used as the extract. For the assay, 2 mL of the extract was combined with 2 mL of DPPH solution, incubated in the dark at 25°C for 30 min, and the absorbance was measured at 517 nm using a spectrophotometer (XD‐7500; Lovibond, Germany).

### 2.11 Baking Loss, Volume, and Density of Pancakes

Baking loss was calculated using the following equation (Shao et al. 2015):

$$\begin{array}{c} \text{Baking loss}\;\left(\%\right)\\ =\left(\text{pancake batter mass before baking}-\text{pancake mass after baking}\right)\\ /\text{pancake batter mass before baking} \end{array}$$

The volume and density of pancakes were measured using the method explained by Amin Ekhlas et al. (2023).

### 2.12 Puncture Test

The hardness of the pancakes was evaluated using the puncture test method with a texture analyzer (Santam, STM‐5, Iran). The test was performed using a cylindrical probe with a diameter of 5 mm, at a constant speed of 0.1 cm/s, and a penetration depth of 1 cm.

### 2.13 Sensorial Attributes of Pancakes

Sensory analysis was performed at the Laboratory of New Technologies at Bu‐Ali Sina University. In this study, twenty‐six panelists (12 men and 14 women) from various age groups including children (5–12 years), adolescents and young adults (14–23 years), adults (25–43 years), and older adults (48–73 years) were recruited to evaluate the pancakes. The indicators considered for sensory evaluation included appearance, odor, flavor, texture, and total acceptance of the pancakes.

### 2.14 Statistical Analysis

All measurements were performed in at least three replicates, and the results were reported as mean ± standard deviation. One‐way ANOVA analysis was performed using SPSS software (ver. 21; SPSS Inc., Chicago, IL, USA). Duncan's multiple range test was used for multiple comparisons, with a significant difference defined at *p* < 0.05.

## 3 Results and Discussion

### 3.1 Color Indexes and Viscosity of Pancake Batter

The effects of flour type on the color indexes of pancake batter were reported in Table 1. Replacing quinoa flour with wheat flour caused the batter to darken in color and decrease in lightness index. The lowest lightness index was found for batter made from sprouted quinoa flour. The redness index of the pancake batters ranged from −6.90 to 0.36 and increased with the substitution of sprouted quinoa flour. There was a considerable difference in the yellowness index between the batters (*p* < 0.05). The yellowness index for pancake batters containing wheat flour, quinoa flour, and sprouted quinoa flour was 24.09, 31.70, and 42.63, respectively.

**TABLE 1:** Effect of flour type on the color indexes of pancake batter.

Flour type	Lightness	Redness	Yellowness
Wheat	89.80 ± 1.32^a^	−6.90 ± 0.18^c^	24.09 ± 0.85^c^
Unsprouted quinoa	85.57 ± 2.64^ab^	−2.66 ± 0.55^b^	31.70 ± 1.15^b^
Sprouted quinoa	84.02 ± 1.88^b^	0.36 ± 0.59^a^	42.63 ± 0.82^a^

*Note:* Data are shown as mean ± standard deviation (*N* = 3). Different letters within columns indicate significant differences (*p* < 0.05).

**FIGURE 2:**
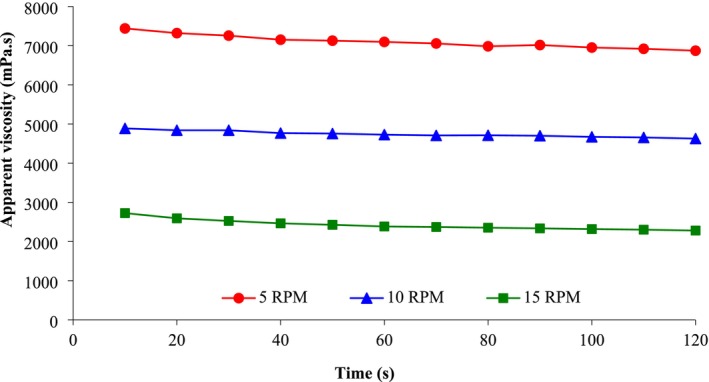
Apparent viscosity of pancake batter containing sprouted quinoa flour as a function of spindle speed (5–15 RMP) and time (s).

**FIGURE 3:**
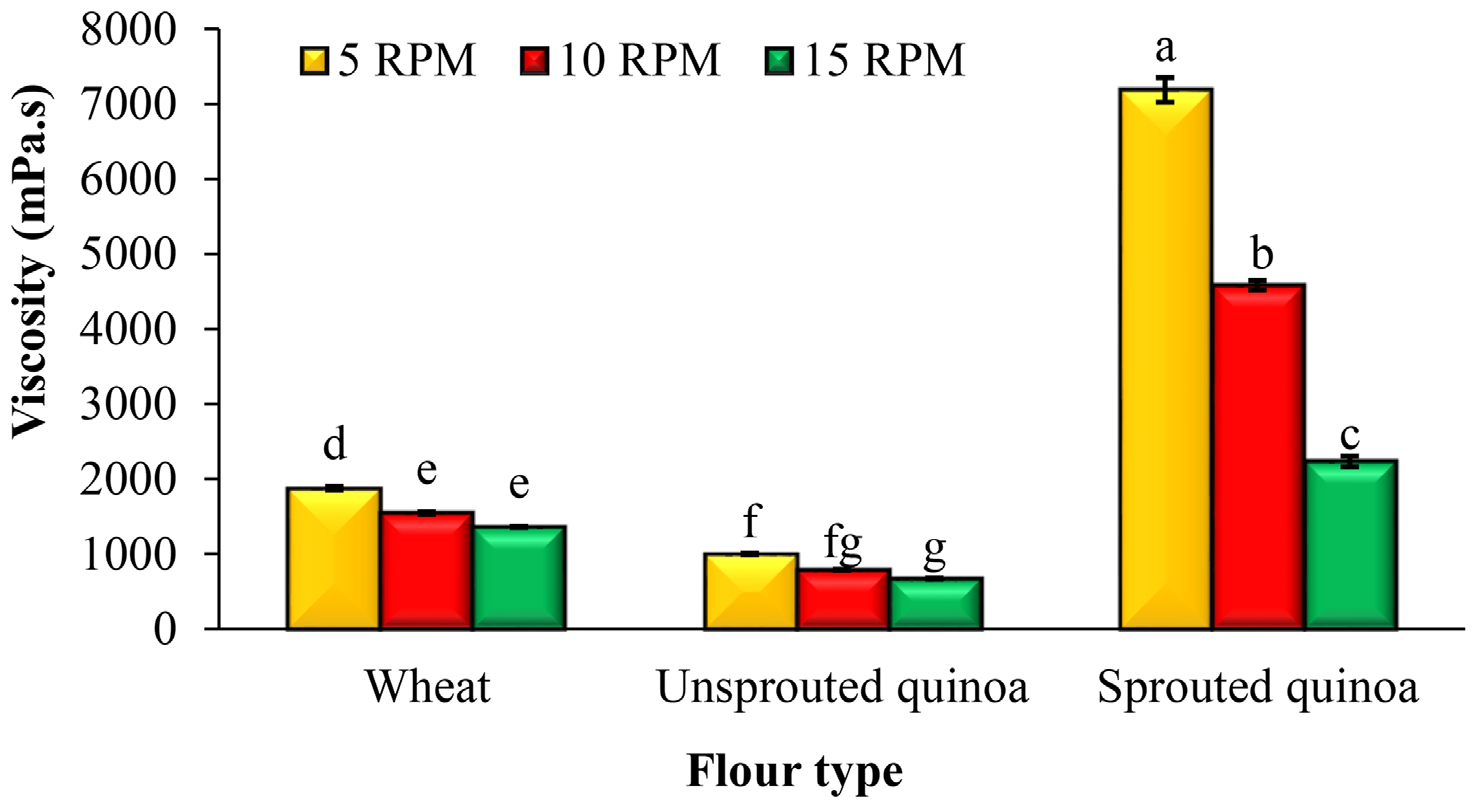
Effect of flour type and spindle revolution speed on the viscosity of pancake batter. Different letters above the columns indicate significant differences (*p* < 0.05).

Figure 2 shows the apparent viscosity of pancake batter as a function of spindle revolution speed and time. As the spindle rotation speed and shear rate increased, the viscosity of the batters decreased, indicating the pseudoplastic behavior of the batters. During the spindle rotation time, the viscosity did not decrease much and the viscosity of the batters was not time‐dependent. Figure 3 shows the influence of flour type and spindle revolution speed on the viscosity of pancake batters. The batter made from sprouted quinoa flour had less flow ability and this batter had a higher viscosity than the other samples. The lowest viscosity was found in batters made from unsprouted quinoa flour.

### 3.2 pH and Acidity of Pancakes

**FIGURE 4:**
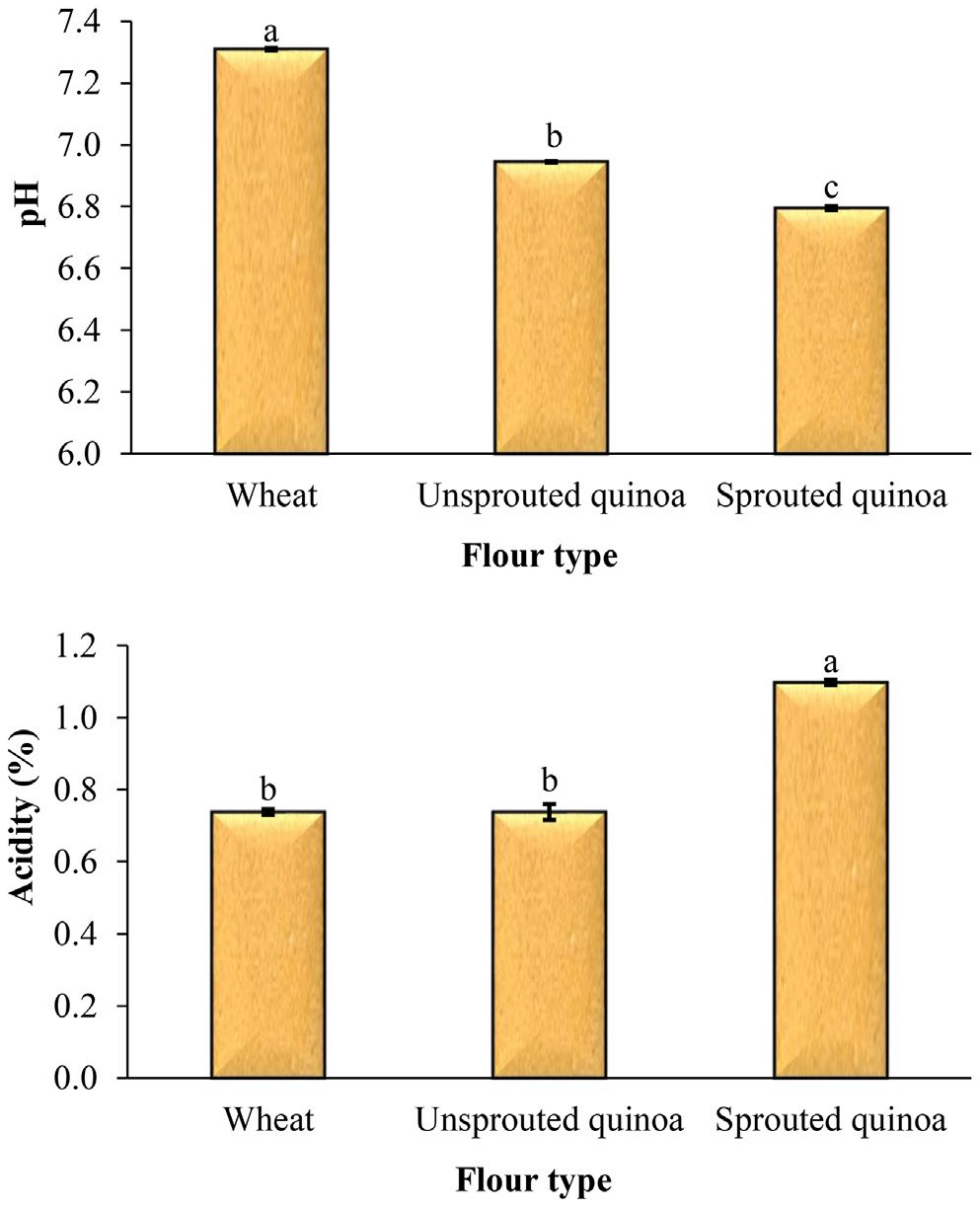
Effect of flour type on the pH and acidity of pancake. Different letters above the columns indicate significant differences (*p* < 0.05).

Figure 4 shows the influence of flour type on the pH and acidity of the pancake. The sprouting process reduces the pH and increases the acidity of sprouted quinoa flour, so the pH and acidity of pancakes made from it were lower and higher than those of other pancakes, respectively. There was a considerable difference in the pH value between the pancakes (*p* < 0.05). The pH values for pancakes containing wheat flour, quinoa flour, and sprouted quinoa flour were 7.31, 6.95, and 6.80, respectively.

During sprouting, the acid content of the quinoa increases due to increased enzymatic and microbial activity. The employ of magnetic field improved the sprouting rate of the quinoa compared to the use of untreated water. As a result, the acidity of the sprouted quinoa flour increases. In this study, there was no significant difference in acidity between pancakes made from wheat flour and quinoa flour (*p* > 0.05). The highest acidity was found in pancakes made from sprouted quinoa flour (1.10), which had a considerable difference from the other two samples (*p* < 0.05).

### 3.3 Core and Crust Color Indexes of Pancakes

Color is an important property of food products. The crust color of baked products is primarily due to the Maillard reaction and caramelization of sugars that occur at baking temperatures. The core and crust color indexes of pancakes are shown in Table 2. When analyzing color indexes, the lightness index or L* ranges from 0 to 100. The closer this number is to 100, the lighter the sample is (Salehi 2019a). In this study, the core and crust lightness index values of pancakes ranged from 68.51 to 77.57 and 51.10 to 56.94, respectively. The pancakes made with wheat flour had the brightest colors for both the inside and outside parts. Javaheripour et al. (2022) reported a decline in the lightness index and darkening of the cakes when using quinoa and germinated wheat flour in sponge cake formulation and attributed the cause to increased enzyme activity and an increase in the amount of reducing sugars. Ozcelik et al. (2024) reported the lightness index of functional and control pancakes in the range of 53.87 to 65.01. The effect of sprouting wheat grains on the color of cupcakes was examined by Ahmed et al. (2024). The results showed that the sprouting of grains resulted in significantly reduced cupcake lightness to 43.17 and 32.73 for 1 and 5 days, respectively, compared with that of the control cupcake (51.79).

**TABLE 2:** Effect of flour type on the core and crust color indexes (lightness, redness, and yellowness) of pancakes.

Flour type	Core color			Crust color		
	Lightness	Redness	Yellowness	Lightness	Redness	Yellowness
Wheat	77.57 ± 0.75^a^	−5.88 ± 0.30^c^	38.06 ± 1.55^b^	56.94 ± 0.45^a^	17.39 ± 0.72^a^	49.18 ± 2.54^a^
Unsprouted quinoa	68.51 ± 1.35^b^	−1.33 ± 0.49^b^	39.06 ± 0.83^b^	52.18 ± 1.85^b^	15.78 ± 1.83^a^	44.01 ± 0.99^b^
Sprouted quinoa	70.05 ± 0.77^b^	1.55 ± 0.18^a^	42.64 ± 0.37^a^	51.10 ± 1.45^b^	18.24 ± 1.99^a^	32.89 ± 1.82^c^

*Note:* Data are shown as mean ± standard deviation (*N* = 3). Different letters within columns indicate significant differences (*p* < 0.05).

The redness index or a* range from −120 to +120, and a negative value of this index indicates that the sample is green, whereas a positive value indicates that the crust color is closer to red (Salehi 2019a). In this study, the core and crust redness index values of pancakes ranged from −5.88 to 1.55, and 17.39 to 18.24, respectively. The minimum value of redness was for the core of pancakes made with wheat flour. Sprouting increases enzymatic activity, resulting in increased enzymatic and nonenzymatic browning reactions, and therefore, increased product redness. The pancakes made with sprouted quinoa flour were redder. The highest value of redness was for the crust of pancakes made with sprouted quinoa flour. According to a report by Ozturk et al. (2014), flour becomes darker than wheat seed flour upon germination, the lightness index decreases and the yellowness and redness indexes increase upon sprouting. Ozcelik et al. (2024) reported the redness index of functional and control pancakes in the range of 12.09 to 13.14.

The yellowness index or b* has a range between −120 and +120, and a negative value means that the crust color is closer to blue, whereas a positive value means that the crust color is closer to yellow (Salehi 2019a). In this study, the core and crust yellowness index values of pancakes ranged from 38.06 to 42.64 and 32.89 to 49.18, respectively. The core yellowness index of pancakes made with sprouted quinoa flour was more intense and noticeably different from other pancakes (*p* < 0.05). At the same time, the crust of pancakes made with wheat flour was also yellower and significantly different from the others (*p* < 0.05). Ozcelik et al. (2024) reported the yellowness index of functional and control pancakes in the range of 27.68 to 31.32.

### 3.4 Total Phenolic Content of Pancakes

**FIGURE 5:**
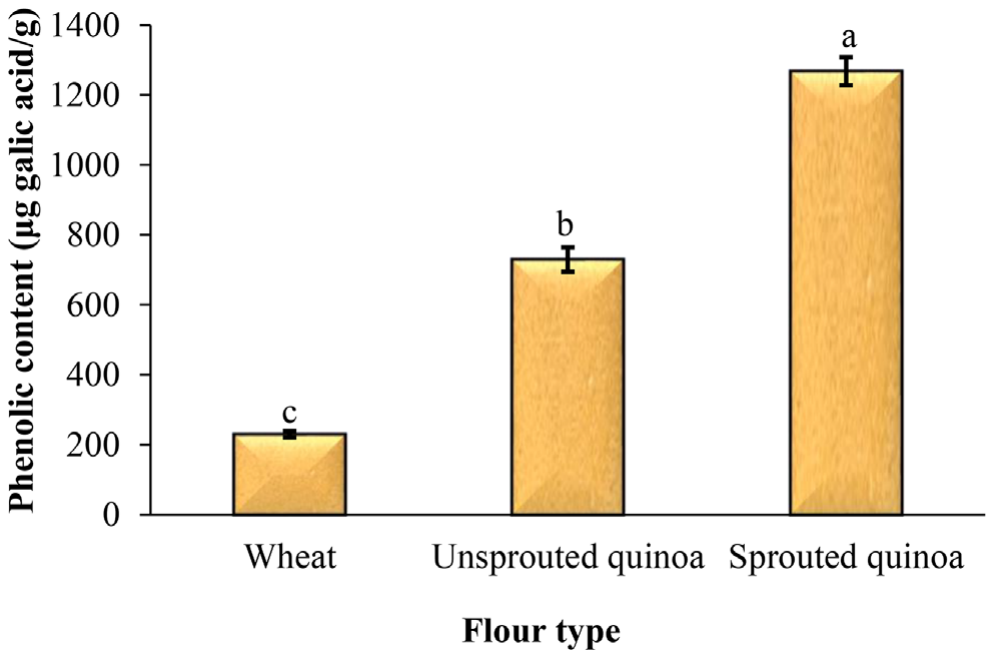
Effect of flour type on the total phenolic content of pancake batter. Different letters above the columns indicate significant differences (*p* < 0.05).

Treatments with magnetized water and a magnetic field increase the sprouting rate, leading to an enhancement in the phenolic compounds in the sprouts and a higher total phenolic content in the sprouted quinoa flour and pancakes. Figure 5 shows the impact of flour type on the total phenolic content of pancakes. The lowest total phenolic content was for the pancakes made with wheat flour (229.70 μg gallic acid/g). The total phenolic content of pancakes made from quinoa flour was higher, and the amount of phenolic compounds increased even more with sprouting. The highest total phenolic content was for the pancakes made with sprouted quinoa flour (1268.17 μg gallic acid/g). Ozcelik et al. (2024) reported the total phenolic content of functional and control pancakes in the range of 313.7–1300.7 μg gallic acid/g. In a study, Cao et al. (2022) examined the effect of sprouted oat flour as a substitute for wheat bread starch on texture and digestibility. They found that the content of polyphenols and butyric acid in the product increased, suggesting that the sprouting process could produce a more digestible and nutritious bread. The aim of the study by Marie et al. (2024) was to evaluate the quality characteristics of black quinoa flour subjected to different pretreatments, including sprouting, roasting, and steaming, as well as to assess the quality attributes of gluten‐free cupcakes formulated with varying substitution levels of the processed quinoa flour. The results demonstrated that the processed quinoa flour exhibited a significantly higher antioxidant capacity compared to the control sample.

### 3.5 Antioxidant Capacity of Pancakes

**FIGURE 6:**
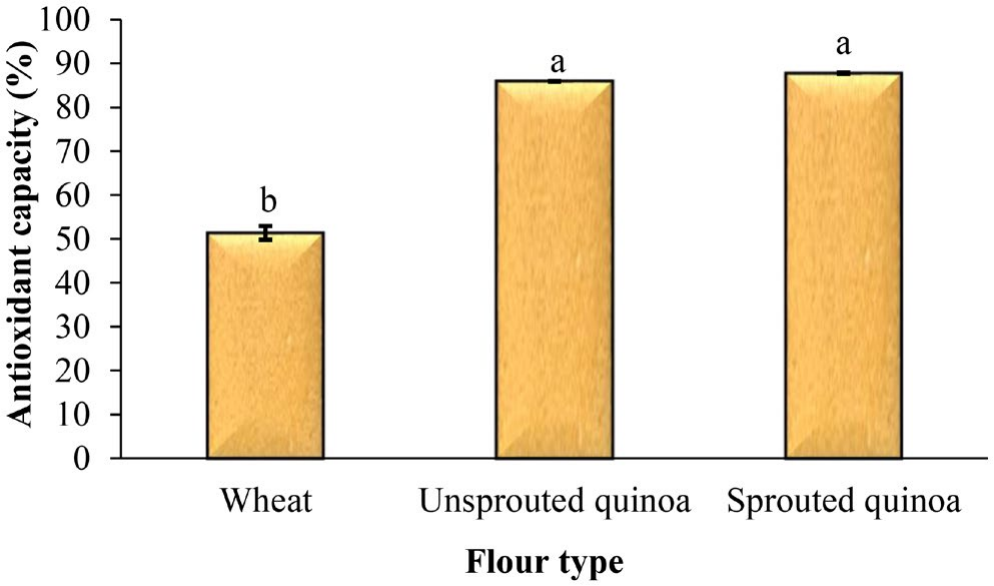
Effect of flour type on the antioxidant capacity of pancake batter. Different letters above the columns indicate significant differences (*p* < 0.05).

The antioxidant capacity is a key bioactive property that has been extensively investigated in germinated grains and edible seeds. Numerous studies have demonstrated that the process of germination significantly enhances the antioxidant capacity of various edible seeds. Figure 6 shows the influence of flour type on the antioxidant capacity of pancakes. This figure shows that the lowest antioxidant capacity was associated with pancakes made with wheat flour and was statistically significant compared to the other pancakes (*p* < 0.05). The antioxidant capacity of pancakes made from quinoa flour was higher, and the antioxidant capacity increased even more with sprouting. The highest antioxidant capacity was for the pancakes made with sprouted quinoa flour (87.77% gallic acid/g). The effect of sprouting wheat grains on the antioxidant capacity of cupcakes was examined by Ahmed et al. (2024). Their results showed that the sprouting of grains for up to 3 days improves nutritional composition and increases antioxidant capacity, and makes suitable and healthier cupcakes. In a study, Paucar‐Menacho et al. (2022) investigated the influence of substituting wheat flour with sprouted cañihua, kiwicha, and quinoa flours on the nutritional composition of biscuits. The study revealed that sprouted pseudocereal flours had reduced starch and protein content, comparable fat, ash, and phytic acid content, and higher concentrations of bioactive compounds and antioxidant capacity compared to wholegrain flours. Notably, sprouted cañihua and quinoa flours positively influenced bioactive compounds and antioxidant capacity in biscuits. The results confirmed the nutritional benefits of incorporation of flours from sprouted Andean grains in the production of baked products.

### 3.6 Baking Loss, Volume, and Density of Pancakes

**FIGURE 7:**
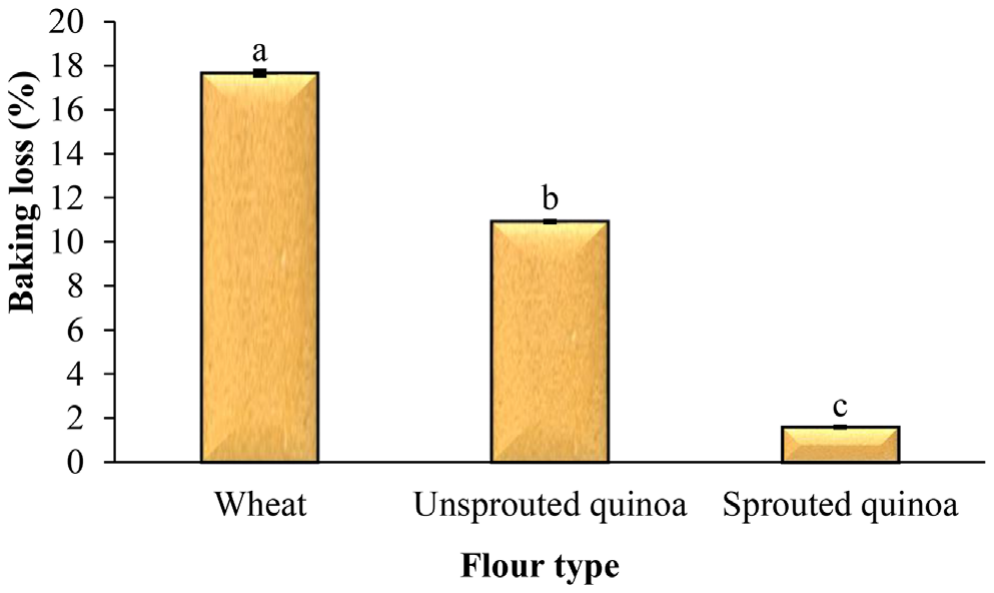
Effect of flour type on the baking loss (%) of pancake. Different letters above the columns indicate significant differences (*p* < 0.05).

The baking loss of pancakes made from wheat flour, quinoa flour, and sprouted quinoa flour was 17.66%, 10.92%, and 1.59%, respectively. The baking loss of pancakes made from sprouted quinoa flour was very low (Figure 7). After cooking, the volume of these pancakes was also much larger than the volumes of the other samples.

**FIGURE 8:**
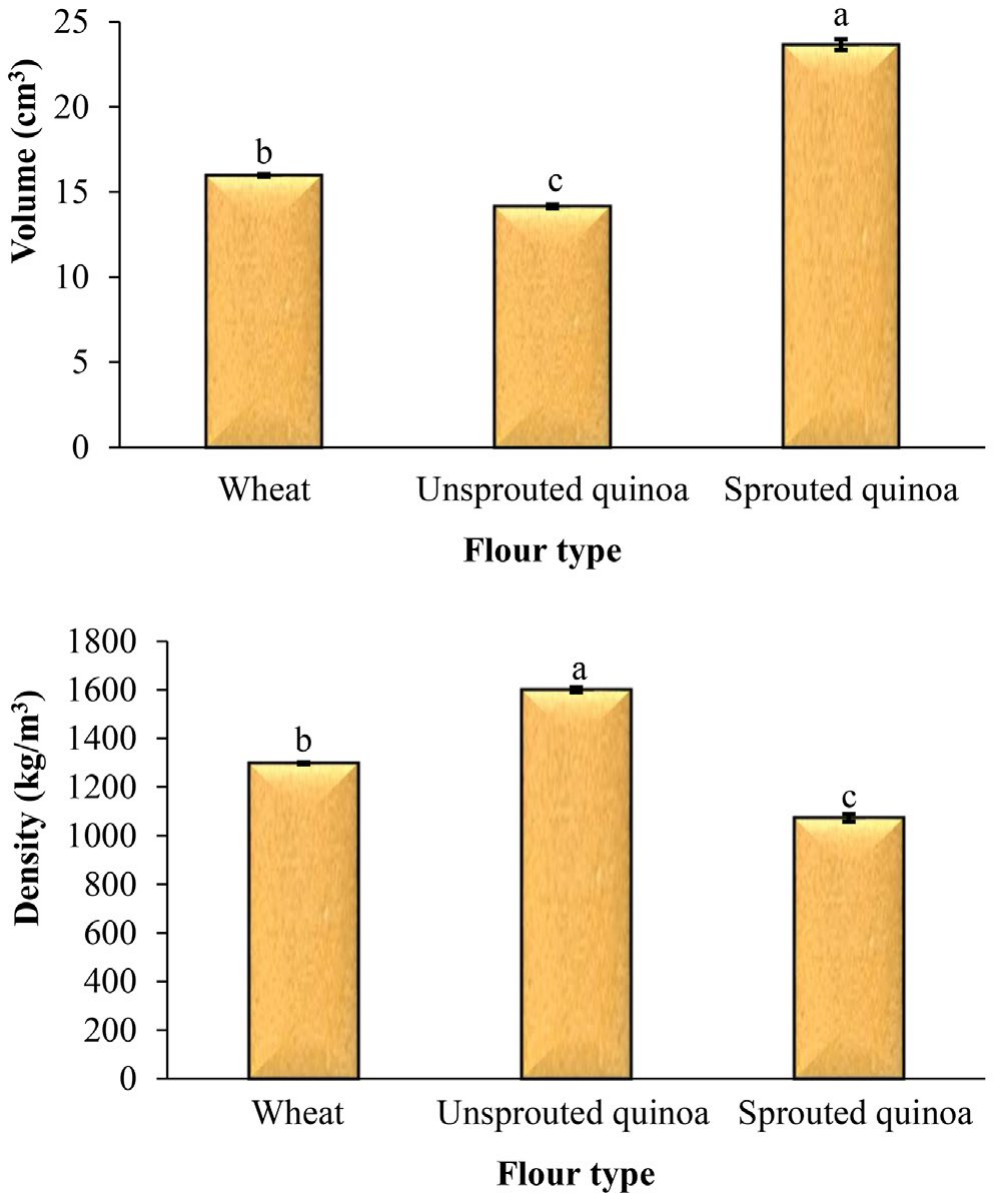
Effect of flour type on the volume and density of pancake. Different letters above the columns indicate significant differences (*p* < 0.05).

The volume is a direct indicator for assessing the quality of pancakes and may reflect the extent of volume expansion and the gas retention ability of the cake or pancake. Figure 8 shows the impact of flour type on the volume of the pancakes made with wheat, quinoa, and sprouted quinoa flour. The results show significant differences in the volume of the pancakes (*p* < 0.05). The highest volume was for the pancakes made with sprouted quinoa flour (23.67 cm^3^). After baking, the volume of pancakes made from quinoa flour was smaller than that made from wheat flour. However, using sprouted quinoa flour solved this problem, and the volume and size of the pancakes improved.

The density of the pancakes is also reported in Figure 8. Significant differences were observed between the density of pancakes made with wheat and quinoa flours (*p* < 0.05). The lowest density was for the pancakes made with sprouted quinoa flour. The density of pancakes made from wheat flour, quinoa flour, and sprouted quinoa flour was 1297.99, 1600.64, and 1072.72 kg/m^3^, respectively.

### 3.7 Hardness of Pancakes

**FIGURE 9:**
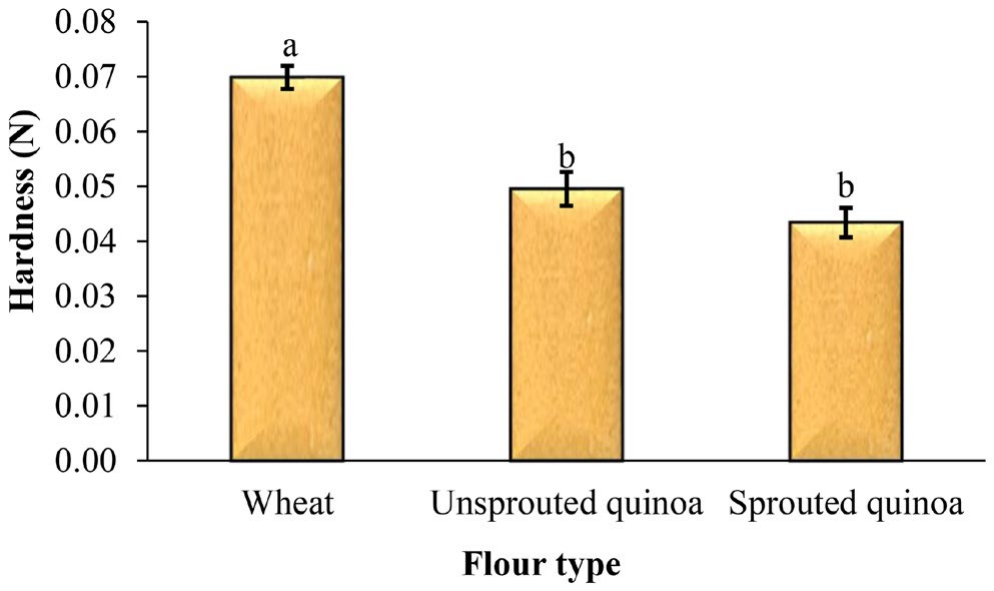
Effect of flour type on the hardness of pancake batter. Different letters above the columns indicate significant differences (*p* < 0.05).

One of the most important quality attributes of food products, including cakes and pancakes, is their texture, and it has a great impact on the total acceptance of the product by the consumer. Figure 9 shows the hardness of the pancakes made with wheat, quinoa, and sprouted quinoa flour. The pancakes prepared with sprouted quinoa flour exhibited the lowest hardness. These pancakes also showed greater volume and lower density compared to the other samples, resulting in a softer texture. The average hardness of pancakes made from wheat flour, quinoa flour, and sprouted quinoa flour was 0.070 N, 0.050 N, and 0.043 N, respectively.

### 3.8 Sensory Evaluation of Pancakes

Table 3 shows the findings of sensory evaluation of pancakes prepared with wheat, quinoa, and sprouted quinoa flours. In terms of appearance, odor, flavor, texture, and total acceptance, there were no considerable differences between the pancakes made from wheat flour and sprouted quinoa flour (*p* > 0.05). According to this table, in most sensory attributes, especially overall acceptance, pancakes made from sprouted quinoa flour received the maximum scores, and pancakes made from unsprouted quinoa flour received the minimum scores. These pancakes had more volume and softness than the other samples and less density and hardness. They had a desirable appearance, color, and taste. Therefore, they received high sensory scores. The satisfaction level of the flavor and texture of pancakes prepared with sprouted quinoa flour was higher, and the reason for this is the sweet taste of quinoa sprouts due to the production of free sugars during the germination process. Pancakes made from quinoa flour received the lowest scores in terms of all sensory attributes and had a statistically significant difference from the other two samples (*p* < 0.05). Suárez‐Estrella et al. (2020) reported that replacing quinoa seeds with sprouted quinoa is an effective approach to improve the characteristics of quinoa‐enriched bread.

**TABLE 3:** Effect of flour type on the sensory attributes of pancakes.

Flour type	Appearance acceptance	Odor acceptance	Flavor acceptance	Texture acceptance	Total acceptance
Wheat	8.6 ± 0.56^a^	8.8 ± 0.42^a^	8.7 ± 0.48^a^	8.8 ± 0.36^a^	8.6 ± 0.49^a^
Unsprouted quinoa	8.2 ± 0.89^b^	7.7 ± 0.98^b^	7.7 ± 0.98^b^	8.3 ± 0.61^b^	7.8 ± 0.77^b^
Sprouted quinoa	8.8 ± 0.42^a^	8.7 ± 0.55^a^	8.6 ± 0.69^a^	8.9 ± 0.27^a^	8.7 ± 0.44^a^

*Note:* Data are shown as mean ± standard deviation (*N* = 26). Different letters within columns indicate significant differences (*p* < 0.05).

## 4 Conclusion

It is possible to make gluten‐free pancakes from quinoa flour. So, in this study, wheat, quinoa seed, and sprouted quinoa seed flours were used to prepare pancakes, and the quality and sensory attributes of products were evaluated. The pancake batter made from sprouted quinoa flour had less flow ability, and this batter had a higher viscosity than the other samples. The sprouting process reduces the pH and increases the acidity of sprouted quinoa flour, so the pH and acidity of pancakes made from this flour were lower and higher than those of other pancakes, respectively. Sprouting increases enzymatic activity, resulting in increased enzymatic and nonenzymatic browning reactions, and therefore, increased product redness. The total phenolic content and antioxidant capacity of pancakes made from quinoa flour were higher, and the amount of phenolic compounds increased even more with sprouting. The baking loss of pancakes made from sprouted quinoa flour was very low, and after cooking, the volume of these pancakes was also much larger than that of the other samples. In addition, these pancakes had more volume and softness than the other samples, and less density and hardness. Overall, considering its high nutritional value, favorable appearance, and acceptable sensory attributes, sprouted quinoa flour is recommended as a functional ingredient for the formulation of various baked products, such as cakes and pancakes. Its incorporation not only enhances the nutritional profile of these products but also supports the development of gluten‐free alternatives with improved health benefits and consumer appeal.

## Author Contributions

**Sepideh Vejdanivahid:** data curation (equal), formal analysis (equal), investigation (equal), methodology (equal), software (equal), writing – original draft (equal). **Fakhreddin Salehi:** conceptualization (equal), data curation (equal), formal analysis (equal), investigation (equal), methodology (equal), project administration (equal), software (equal), supervision (equal), validation (equal), writing – original draft (equal), writing – review and editing (equal).

## Ethics Statement

The authors have nothing to report.

## Conflicts of Interest

The authors declare no conflicts of interest.

## Data Availability

All data generated or analyzed during this research are included in this published manuscript.
